# Divergent Immune–Metabolic Profiles in Endometriosis and Ovarian Cancer: A Cross-Sectional Analysis

**DOI:** 10.3390/cancers17142325

**Published:** 2025-07-12

**Authors:** Manuela Neri, Elisabetta Sanna, Paolo Albino Ferrari, Clelia Madeddu, Eleonora Lai, Valerio Vallerino, Antonio Macciò

**Affiliations:** 1 Department of Obstetrics and Gynecological Oncology, Azienda di Rilievo Nazionale ed Alta Specializzazione “G. Brotzu”, 09121 Cagliari, Italy; manuela.neri@aob.it (M.N.); elisabetta.sanna@aob.it (E.S.); valerio.vallerino@aob.it (V.V.); antoniopm.maccio@aob.it (A.M.); 2 Department of Oncological Surgery, Azienda di Rilievo Nazionale ed Alta Specializzazione “G. Brotzu”, 09121 Cagliari, Italy; 3 Department of Oncological Science and Public Health, University of Cagliari, SS 554 km 4.500, 09042 Monserrato, Italy; clelia_md@yahoo.it (C.M.); ele.lai87@gmail.com (E.L.); 4 Department of Surgical Science, University of Cagliari, SS 554 km 4.500, 09042 Monserrato, Italy

**Keywords:** endometriosis, ovarian cancer, macrophage polarization, iron metabolism, oxidative stress

## Abstract

Background/Objectives: Endometriosis and high-grade serous ovarian cancer (HGS-OC) share common features within the peritoneal immune microenvironment, yet they exhibit divergent clinical outcomes. This study aimed to dissect the immune–metabolic landscape of the peritoneal cavity in patients with endometriosis and ovarian cancer by evaluating macrophage polarization, intracellular signaling pathways, and iron-driven oxidative stress. Methods: A prospective cohort study enrolled 40 patients with endometriosis, 198 with ascitic ovarian cancer (178 HGS-OC), and 200 controls with benign gynecological conditions. Peritoneal and peripheral blood samples were analyzed via flow cytometry for macrophage (M1/M2) polarization markers, mTOR/AKT expression, and glucose uptake. Inflammatory markers (IL-6, CRP), oxidative stress (ROS), and iron metabolism parameters (hepcidin, ferritin, transferrin, serum/free iron) were quantified. Results: HGS-OC displayed a predominance of M1-polarized tumor-associated macrophages (TAMs) (CD14⁺/CD80⁺/Glut1⁺) and a high M1/M2 ratio (2.5 vs. 0.8 and 0.9; *p* = 0.019), correlating positively with IL-6 (*p* = 0.015), ROS (*p* = 0.023), hepcidin (*p* = 0.038), and ferritin (*p* = 0.043). Conversely, endometriosis showed a dominant M2 profile (CD14⁺/CD163⁺), elevated intracellular mTOR and AKT expression in both TAMs and epithelial cells (*p* < 0.01), and significantly higher ascitic ROS and free iron levels (*p* = 0.047 and *p* < 0.0001, respectively). In endometriosis, the M1/M2 ratio correlated inversely with free iron (*p* = 0.041), while ROS levels were directly associated with iron overload (*p* = 0.0034). Conclusions: Endometriosis exhibits a distinct immune–metabolic phenotype characterized by M2 macrophage predominance and iron-induced oxidative stress, contrasting with the inflammatory, M1-rich profile of HGS-OC. These findings suggest that iron metabolism and macrophage plasticity contribute to disease persistence in endometriosis and may inform future immunomodulatory strategies.

## Simple Summary

Endometriosis and ovarian cancer display contrasting immune–metabolic profiles. Endometriosis is associated with an M2-polarized, iron-rich, pro-oxidative environment, suggesting macrophage-driven immune tolerance. These findings highlight the potential of targeting macrophage phenotypes and iron metabolism in managing endometriosis and warrant further investigation.

## 1. Introduction

Endometriosis is a chronic inflammatory disease affecting approximately 10–20% of women of reproductive age and is characterized by the ectopic implantation of endometrial-like tissue outside the uterine cavity. Clinically, it is associated with pelvic pain, infertility, and significant impairment in quality of life [1].

Although retrograde menstruation remains the leading hypothesis for its pathogenesis [2], increasing evidence supports the role of immune dysfunction and a permissive microenvironment in promoting ectopic implantation and persistence of endometrial tissue [3]. Aberrant immune responses within the peritoneal cavity can facilitate lesion survival, angiogenesis, and resistance to immune clearance [4].

Recent studies have highlighted the contrasting immune profiles between endometriosis and malignancies such as high-grade serous ovarian cancer (HGS-OC), particularly in the peritoneal environment [5]. Flow cytometric analyses have demonstrated a predominance of alternatively activated (M2) macrophages in endometriosis, resulting in a low M1/M2 ratio and promoting an iron-rich, oxidative, and fibrotic environment [6,7]. Conversely, HGS-OC typically displays a higher abundance of classically activated (M1) macrophages associated with heightened immune activity, increased chemosensitivity, and improved prognosis [8].

The tumor or lesion microenvironment, encompassing immune cells, stromal elements, the extracellular matrix, vascular structures, and soluble mediators, plays a central role in regulating inflammation, tissue remodeling, and immune escape [9,10,11]. This niche often promotes immune evasion in neoplastic tissues through cell–cell interactions and soluble factors that reprogram immune cells [12,13,14,15].

**Figure 1:**
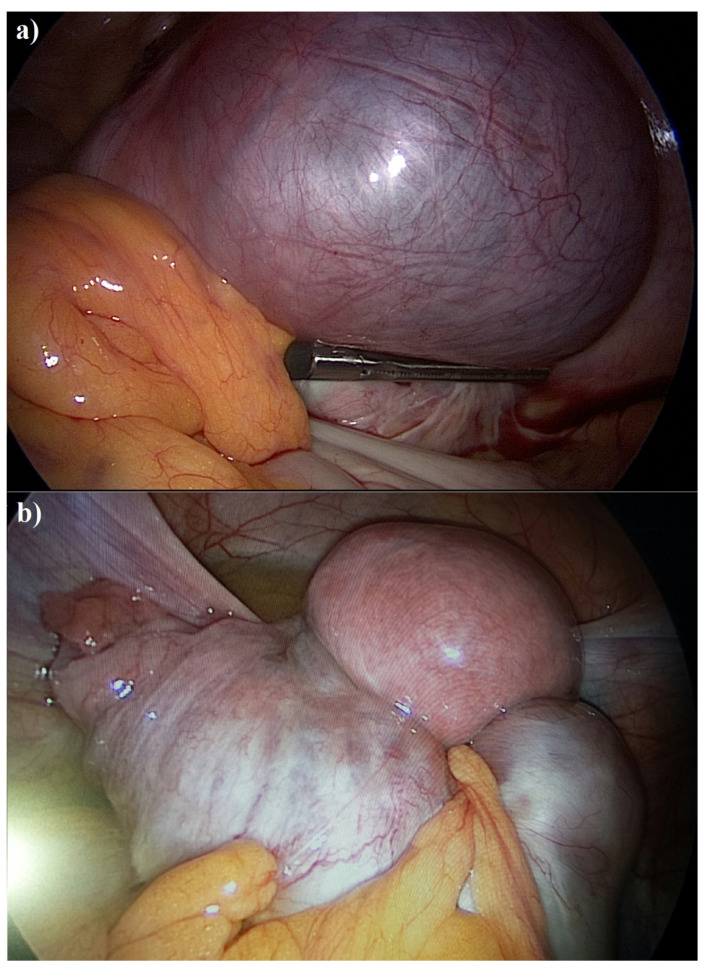
(**a**) Laparoscopic grasper holds the adhesion between a large endometrioid bilateral cyst of the left ovary and the lateral wall of the pelvic cavity. Adhesions were thick/dense and showed multiple hemorrhagic spots. (**b**) Laparoscopic view showing a large, smooth-surfaced, right ovarian mass with tense cystic morphology and prominent subserosal vascularization, resulting in right-sided endometrioid ovarian carcinoma. (Obstetrics and Gynecological Oncology, A.R.N.A.S. “G. Brotzu”, Cagliari, Italy).

Despite its benign nature, endometriosis exhibits several neoplasm-like features, including invasion, dissemination, resistance to apoptosis, and immune evasion [16,17]. Macroscopically, endometriomas can resemble endometrioid ovarian carcinomas (Figure 1), yet their pathobiology and clinical outcomes remain distinct [18].

Within the peritoneal environment, M2 macrophages and regulatory T cells (Tregs) contribute to immune tolerance and lesion persistence in endometriosis [19], a phenomenon that mirrors the role of tumor-associated macrophages (TAMs) in cancer, where high M2 density correlates with a poor prognosis [20]. Immunotherapeutic strategies targeting TAMs, via depletion or reprogramming toward the M1 phenotype, are under investigation in both cancer and chronic inflammatory diseases [21].

Additionally, the immunoscore, a metric based on tumor-infiltrating lymphocytes, has emerged as a prognostic marker in oncology and may hold translational relevance for immune profiling in endometriosis [22].

This study was designed to characterize and compare the peritoneal immune landscapes in endometriosis and ovarian cancer. We focused on macrophage polarization, mTOR/AKT signaling, and iron metabolism to elucidate immune–metabolic mechanisms that may underlie their divergent clinical and biological behavior.

## 2. Materials and Methods

We conducted a prospective observational cohort study involving patients diagnosed with endometriosis who met surgical criteria and patients with ascites secondary to advanced primary OC (stage IIIC–IV). All subjects were referred to the Gynecologic Oncology Unit at Businco A.R.N.A.S. Brotzu Hospital between January 2020 and January 2024. The study also included a control group of 200 patients undergoing surgery for benign gynecologic conditions (e.g., fibroids, benign ovarian cysts), from whom peritoneal washings were collected intraoperatively.

Inclusion criteria included the presence of ovarian endometriotic lesions with extensive adhesions and deep pelvic involvement (e.g., rectosigmoid, vesicouterine fold, parietal peritoneum).

Patients with endometriosis were evaluated during laparoscopic surgery. Due to suspect thoracic endometriotic implants, pleural effusion patients were preoperatively assessed by a thoracic surgeon for thoracoscopic exploration and diaphragmatic resection. Patients with OC were also assessed laparoscopically before receiving any systemic therapy.

Laparoscopy was the standard surgical approach due to its advantages in terms of reduced postoperative pain, shorter hospitalization, quicker recovery, and better cosmetic results [23].

In accordance with the 2022 ESHRE guidelines [24], patients who had completed childbearing and exhibited resistance to conservative management underwent hysterectomy with bilateral salpingo-oophorectomy and resection of all visible endometriotic lesions. Surgical strategy and extent were personalized based on disease burden, symptom severity, side effects, and patient preferences.

The study protocol was conducted in accordance with the Declaration of Helsinki. Informed consent was obtained from all participants for surgical treatment, study participation, and biological sample collection.

### 2.1. Measured Parameters

All enrolled patients underwent peripheral blood sampling and peritoneal/ascitic fluid collection. Lymphocytic and macrophagic cells in the ascitic fluid were isolated, and their functional/metabolic phenotype and polarization status were assessed following previously described methodologies [25]. The following parameters were evaluated in peripheral blood: serum levels of IL-6, CRP, fibrinogen, hepcidin, ferritin, transferrin, free iron, and blood levels of ROS. The following parameters were evaluated in the peritoneal fluid: lymphocyte and TAM phenotype, intracellular expression of mTOR and AKT, ascitic levels of IL-6, hepcidin, ferritin, transferrin, free iron, and ROS.

### 2.2. Isolation of Lymphocyte Populations and TAMs from Ascitic Fluid

Immediately after collection, ascitic fluid was centrifuged at 300× *g* for 10 min at room temperature (RT) to obtain a cellular pellet. Supernatants were stored at −80 °C until analysis. Lymphocyte populations and tumor-associated macrophages (TAMs) were isolated using a double-layer Ficoll–Hypaque density gradient (100% and 75%, respectively; Fresenius Kabi, Morge AS, Oslo, Norway). The separation was performed by centrifugation at 300× *g* for 30 min at room temperature. TAMs were then harvested, washed twice with Hank’s Balanced Salt Solution (HBSS; GIBCO, Carlsbad, CA, USA), and counted. Cell viability was assessed using the Trypan Blue dye exclusion assay.

### 2.3. Characterization of Lymphocyte/Macrophage Subsets and TAM Polarization

Immediately following cell separation, lymphocyte phenotyping was performed using fluorescein isothiocyanate (FITC)-conjugated monoclonal antibody (mAb) against CD3, as well as dual labeling with phycoerythrin (PE)-conjugated anti-CD4 and anti-CD8 mAbs. The CD4/CD8 ratio was then calculated and compared across disease groups. Tumor-associated macrophages (TAMs) were identified using anti-CD14 mAb. Flow cytometric analysis of CD14 expression revealed that CD14⁺ cells represented the predominant monocyte population (86.8%), with fewer than 20% non-lymphomonocytic cells and over 90% viability. TAMs were selected based on forward scatter and side scatter properties.

All data were acquired using a FACScan flow cytometer (Becton Dickinson, Franklin Lakes, NJ, USA) and analyzed with CellQuest software (Version 5.1, BD Biosciences, San Jose, CA, USA). To determine M1/M2 macrophage polarization, cells were stained with FITC-labeled anti-CD14 and PE-labeled anti-human CD80 (M1 marker) or PE-labeled anti-human CD163 (M2 marker, Miltenyi Biotec GmbH, Bergisch Gladbach, Germany), along with either anti-human Glut-1 (Abcam, Cambridge, MA, USA) or anti-HLA-DR (BD Biosciences). Additional markers included APC-labeled anti-CD206 (BD Biosciences) and intracellular APC-labeled anti-Arginase-1 (R&D Systems, Minneapolis, MN, USA).

For surface staining, 100 µL of cell suspension (1 × 10^6^ cells/mL) was incubated with 5–10 µL of each mAb for 15 min at 4 °C, followed by two PBS washes and fixation in 1% paraformaldehyde. For intracellular staining, surface-stained cells were fixed with BD Cytofix™ Buffer for 10 min, washed, permeabilized for 30 min using BD Phosphoflow Perm Buffer III (BD Biosciences, San Jose, CA, USA), and stained with the appropriate intracellular antibody.

To assess functional activation and metabolic characteristics of M1-polarized TAMs, glucose uptake was measured using the fluorescent analog 2-[N-(7-nitrobenz-2-oxa-1,3-diazol-4-yl)amino]-2-deoxy-D-glucose (2-NBDG), with a cell-based glucose uptake assay kit (Cayman Chemical, Ann Arbor, MI, USA). TAMs were suspended at a concentration of ≥5 × 10^5^ cells/mL and incubated with 100 µL of glucose-free medium containing 150 µg/mL of 2-NBDG for 10 min. After incubation, cells were centrifuged at 400× *g* for 5 min at room temperature; the supernatant was discarded, and cells were washed twice with assay buffer. Flow cytometric analysis was performed immediately using a FACScan cytometer and ModFit software (Version 3.3, BD Biosciences). Results were expressed as both the percentage of positive cells and mean fluorescence intensity (MFI).

### 2.4. Intracellular Expression of mTOR and AKT

For the analysis of intracellular levels of mTOR and AKT, cells were labeled with monoclonal anti-mTOR PE and anti-AKT PE antibody (BD Biosciences), double-labeled with anti-CD14 FITC antibody for identification of the macrophage cell component or with anti-EpCAM FITC antibody for identification of the epithelial cell component, using the protocol for intracellular labeling described in the previous paragraph. The antibody used for mTOR analysis binds to the protein’s phosphorylated (activated) form.

### 2.5. Inflammatory Markers, ROS, and Iron Metabolism Parameters in Peripheral Blood and Ascitic Fluid

At the time of enrollment, in all patients, circulating levels of markers of inflammation such as IL-6, C-reactive protein (CRP), and fibrinogen, the reactive oxygen species (ROS), and the parameters of iron metabolism (i.e., hepcidin, ferritin, transferrin, and free iron) were examined. The peritoneal effusion also measured levels of IL-6, ROS, hepcidin, ferritin, transferrin, and free iron. Ferritin, transferrin, free iron, CRP, and fibrinogen were analyzed using the same standard laboratory procedures, in accordance with internal quality control, both in peripheral blood and in peritoneal fluid. IL-6 and hepcidin were evaluated with kits (ELISA) (DRG, Marburg, Germany) with the same protocol, as indicated by the manufacturer, for blood and peritoneal fluid analysis. ROS levels were studied using a colorimetric method (FORT test, Callegari Spa, Parma, Italy), according to the manufacturer’s instructions, for both blood and peritoneal fluid analysis.

### 2.6. Statistical Analysis

Data were reported as mean ± standard deviation (SD) for continuous variables and percentages for categorical variables. Differences between means were examined with the two-tailed, unpaired *t*-test for variables with normal distribution or the Mann–Whitney test for variables with non-normal distribution. Differences between multiple groups were analyzed using ANOVA for normally distributed variables (or Kruskal–Wallis for non-normal variables) and the Tukey–Kramer test for paired comparisons using Bonferroni correction. Regression analysis assessed the association between the various variables, particularly between the M1/M2 ratio and the parameters of inflammation, oxidative stress, and iron metabolism. All tests were two-tailed; a *p* < 0.05 level was considered significant. Statistical analyses were performed using the MedCalc program version 20.115 (2022 MedCalc Software Ltd., Ostend, Belgium).

## 3. Results

From January 2020 to January 2024, we analyzed 40 patients with endometriosis with surgical criteria and 198 consecutive patients with ascites resulting from primary OC (stage IIIC–IV), including 178 patients with HGS-OC and 20 patients with other primary OC histotypes (clear cell and endometrioid) (Table 1).

As for the surgical approach of OC patients, 70% underwent, first, cytoreductive surgery with radical intent (optimal cytoreductive surgery). Among them, 25% underwent one or more bowel resections, 5% received splenectomy, and 70% underwent parietal or total peritonectomy (including diaphragmatic peritonectomy). The remaining patients (30%) were not candidates for primary radical surgery and underwent diagnostic laparoscopy with minimal surgery (isolated omentectomy, adnexectomy, or peritoneal biopsy) to obtain a pathological diagnosis. After surgery, all patients with ovarian cancer received platinum-based chemotherapy according to standard guidelines.

### 3.1. Analysis of Lymphocyte Subpopulations and Tumor-Associated Macrophage Polarization in Ascitic Fluid

The analysis of lymphocyte subpopulations in ascitic fluid demonstrated a significantly lower CD4/CD8 ratio in HGS-OC patients than in other histotypes and endometriosis (*p* = 0.016) (Table 2). The analysis of macrophage polarization showed that TAMs isolated from ascites of primary HGS-OC are represented mainly by M1 macrophages (CD14+/CD80+/Glut1+ cells) with a higher M1/M2 ratio than patients with endometriosis and other OC histotypes, such as endometrioid and clear cells (2.5 ± 0.7 vs. 0.8 ± 0.3 vs. 0.9 ± 0.4, respectively, *p* = 0.019). The percentage of M1 cells (CD14+/CD80+/Glut+) was significantly lower in endometriosis and in other OC histotypes than in serous papillary carcinoma (23.7 ± 7.9 and 28.6 ± 10.8 vs. 56.7 ± 12; *p* = 0.038) and the percentage of M2 cells (CD14+/CD163+) was significantly higher in endometriosis and in other OC histotypes than in HGS-OC (49.9 ± 11.8 and 52.7 ± 15 vs. 34 ± 11; *p* = 0.047) (Table 2).

### 3.2. Analysis of Intracellular Expression of mTOR, AKT, and PTEN in TAMs and Epithelial Cells Isolated in the Ascitic Fluid

Regarding the intracellular markers of the metabolic mTOR pathway, we found that the intracellular levels of mTOR and AKT in macrophages (CD14+) isolated from the ascitic fluid were significantly higher in endometriosis than in papillary serous ovarian cancer (*p* = 0.001 and *p* = 0.002, respectively). Notably, the intracellular expression of these proteins was comparable to that detected in epithelial cells isolated from ascites (Table 2).

### 3.3. Analysis of Markers of Inflammation, ROS, and Iron Metabolism in Peripheral Blood and Ascitic Fluid

The analysis of circulating inflammation parameters demonstrated significantly lower levels of IL-6, fibrinogen, and CRP in patients with endometriosis than in those with HGS-OC and other OC histotypes (Table 3). Consistently, the circulating levels of hepcidin, ferritin, and ROS were significantly lower in patients with endometriosis than with HGS-OC and other OC histotypes. Serum iron levels were significantly lower in HGS-OC patients than in women with endometriosis and other histotypes (Table 3).

The analysis of these parameters in the ascitic fluid demonstrated that, according to macrophage polarization, IL-6 levels were significantly higher in the ascites of patients with HGS-OC than in those with other OC histotypes and endometriosis (Table 3). According to the evidence that M1 polarization is associated with a shift toward an altered iron metabolism, ascites levels of ferritin and hepcidin were significantly higher in HGS-OC than in those with other OC histotypes and endometriosis. Vice versa, free iron levels were significantly higher in the ascitic fluid of endometriosis patients than in women with HGS-OC (Table 3). ROS levels in the ascites associated with endometriosis were significantly higher than in the ascites of HGS-OC (Table 3).

Thus, we correlated the levels of inflammation, oxidative stress, and iron metabolism parameters in the ascitic fluid with the M1/M2 ratio. We found that the M1/M2 ratio was significantly and directly related to IL-6, hepcidin, ferritin, and ROS levels in OC patients but not in women diagnosed with endometriosis. In patients with endometriosis, a statistically significant inverse correlation was identified between the M1/M2 ratio and free iron levels (Table 4).

In patients with endometriosis, ascitic ROS levels were directly and significantly associated with free iron concentrations, as confirmed by both correlation and linear-regression analyses. In contrast, among ovarian carcinoma cases, ascitic ROS levels showed a significant positive correlation with IL-6 concentrations (Table 4 and Table 5).

## 4. Discussion

Consistent with our previous reports [26], we identified a predominant M2 phenotype (CD14⁺/CD163⁺) in the peritoneal fluid of patients with endometriosis. In contrast, a significantly higher M1/M2 ratio was observed in ascitic fluid from patients with high-grade serous ovarian cancer (HGS-OC) [27,28,29]. M2 macrophages in endometriosis were associated with chronic inflammation, fibrosis, and lesion persistence, contributing to infertility and pelvic adhesions [30]. In contrast, the enrichment/predominance of M1 macrophages (CD14⁺/CD80⁺) in HGS-OC, known for their anti-tumorigenic activity, may enhance chemotherapeutic responsiveness [31]. These findings underscore the pivotal immunomodulatory role of macrophages in defining disease behavior [32].

Our comparative analysis extended to adaptive immune markers and cytokine profiles across disease groups. M2 predominance in endometriosis and non-serous ovarian cancer histotypes aligns with regenerative and immune-evasive characteristics of the tissue microenvironment [33], while the elevated M1/M2 ratio in HGS-OC suggests an immune-activated microenvironment [27].

Endometriosis pathogenesis involves multiple mechanisms, including retrograde menstruation [34], mesothelial metaplasia [35], and embryonic remnants [36]. Estrogen-driven proliferation, Vascular Endothelial Growth Factor (VEGF)-mediated angiogenesis, and immune dysregulation contribute to lesion survival [37]. Immune dysfunction in endometriosis is also supported by the alterations in peritoneal immune cell populations—macrophages [38,39], neutrophils, dendritic cells [40], natural killer cells [41], and Th17/Treg imbalances [42].

Hypoxia and iron overload due to retrograde menstruation may activate peritoneal macrophages, influencing polarization and function [43,44]. This plasticity, driven by local stimuli, results in the emergence of M1 or M2 phenotypes with distinct transcriptomic signatures [45,46].

Despite inherent limitations that should be considered when interpreting the findings, this study offers a comprehensive comparative assessment of the peritoneal immune microenvironment in endometriosis and ovarian cancer. First, its cross-sectional design precludes longitudinal assessment of immune and metabolic shifts over time, limiting causal inference regarding disease progression. Second, the relatively small sample size, particularly in the endometriosis and non-HGS-OC subgroups, may reduce statistical power and limit the generalizability of subgroup analyses. Additionally, while flow cytometry provided detailed phenotypic and metabolic profiling of immune cells, functional assays to validate macrophage activity (e.g., phagocytosis, cytokine secretion) were not performed, restricting mechanistic insight.

Our data revealed significantly higher intracellular mTOR and AKT expression in TAMs (CD14⁺) and epithelial cells from endometriosis patients compared to HGS-OC. This metabolic profile suggests a distinct activation state in endometriosis, potentially mediated by mTOR-driven autocrine and paracrine signaling [47]. It may also reflect a convergence of immune phenotype and metabolic reprogramming in sustaining lesion persistence [48].

Systemic inflammatory markers (IL-6, CRP, fibrinogen, hepcidin, ferritin, and ROS) were markedly lower in endometriosis, consistent with a localized inflammatory process. However, the peritoneal compartment exhibited a distinct immune–metabolic profile: IL-6, hepcidin, and ferritin levels were higher in HGS-OC, whereas ROS and free iron were elevated in endometriosis. This suggests that a pro-oxidant peritoneal environment in endometriosis may sustain lesion development through iron-driven ROS production [49].

Correlation analysis revealed that, in HGS-OC, the M1/M2 ratio was positively correlated with IL-6, hepcidin, ferritin, and ROS, indicating an inflammatory, immune-activated profile. In contrast, in endometriosis, the M1/M2 ratio was inversely associated with free iron, suggesting that iron overload may promote M2 polarization. Moreover, ROS levels correlated directly with free iron, reinforcing the link between oxidative stress, iron metabolism, and immune modulation [50].

Angiogenesis is critical in the progression of both endometriosis and OC. In our study, elevated intracellular mTOR/AKT expression in TAMs from endometriosis patients may reflect a proangiogenic phenotype, consistent with the presence of M2d-like macrophages. These cells secrete VEGF, FGF, and matrix metalloproteinases, which contribute to neovascularization and tissue invasion [7]. The chronic hypoxic and iron-rich microenvironment of endometriotic lesions may further favor this phenotype, as shown by the strong correlation between ROS, free iron, and M2 polarization.

Macrophage dysfunction contributes to peritoneal pathology, including inflammation and adhesion formation [51,52]. In endometriosis, altered macrophage function plays a central role in immune evasion and tissue remodeling [30]. The loss of physiological cyclic variation in macrophage populations within the eutopic endometrium may facilitate the establishment of ectopic lesions [52,53].

Animal studies confirm that macrophage depletion reduces lesion formation, while disrupted monocyte recruitment exacerbates disease burden [54]. Additionally, endometriosis-associated macrophages exhibit a hyperinflammatory phenotype, and in vivo models support that M2 polarization facilitates vascularization and lesion expansion, while M1 polarization exerts suppressive effects [55,56].

A different immune profile and M1/M2 polarization accompany the progression of endometriosis into different stages. In detail, a meta-analysis of available public data showed that activated dendritic cells, CD4 T cells, eosinophils, and M1 macrophages were elevated in stage I–II endometriosis. In contrast, M2 macrophages were elevated in stage III–IV endometriosis [57].

Although benign, endometriosis shares cancer-like properties, including invasiveness and local spread [58]. TAMs promote tumor progression and immune evasion [59,60]. Notably, M1-predominant environments, such as those found in HGS-OC, are associated with improved outcomes and platinum sensitivity [61], whereas M2 polarization correlates with treatment resistance [17].

Moreover, the distinct immune and metabolic profiles in endometriosis and HGS-OC promote the pathogenesis and progression into two different diseases. We can hypothesize that M2 prevalence in endometriosis, differently from HGS-OC, may play a role in halting the evolution toward malignancy by inhibiting the onset of inflammation-induced mutations/atypia and the metastatic process by promoting fibrosis and tissue development [62].

The immune tolerance observed in endometriosis resembles mechanisms in cancer, supporting the hypothesis that a permissive peritoneal environment enables lesion persistence. Understanding these immune microenvironmental divergences could inform novel immunomodulatory strategies to restore immune balance and improve disease outcomes.

## 5. Conclusions

This study demonstrates distinct immune–metabolic signatures in the peritoneal environment of patients with endometriosis compared to those with HGS-OC. The predominance of M2 macrophages, elevated free iron, and increased ROS in endometriosis contrast with the pro-inflammatory and M1-skewed profile of HGS-OC, underscoring divergent immunological and metabolic landscapes. These findings support the hypothesis that macrophage polarization and iron-driven oxidative stress contribute to lesion persistence in endometriosis and may inform future immunomodulatory strategies. Further research integrating longitudinal data and functional validation is warranted to elucidate the mechanistic underpinnings of these observations and to explore potential therapeutic targets.

## Figures and Tables

**Table 1:** Clinical characteristics of enrolled patients.

		Patients with Ovarian Carcinoma	Patients with Endometriosis
Patients enrolled, No.		198	40
Median age, years (range)		64 ± 7 (58–78)	30 ± 10 (19–42)
Median height, cm (range)		160 ± 10 (154–174)	164 ± 15 (150–173)
Median weight, kg (range)		62 ± 15 (37–75)	55 ± 10 (46–68)
Histotype, No. (%)			
	High-grade serous	178 (89.8%)	
	Clear cell	10 (5.1%)	
	Endometrioid	10 (5.1%)	
Stage, No. (%)			
	IIIC	146 (73.7%)	
	IV	52 (26.3%)	

Abbreviation: No., number; cm, centimeters; kg, kilograms.

**Table 2:** Analysis of lymphocyte subpopulations, macrophage phenotypes, and mTOR/AKT intracellular expression in the peritoneal fluid of patients with ovarian cancer and endometriosis.

Expression of Markers of TAM Polarization	HGS-OC No. 178	Other Histotypes No. 20	Endometriosis No. 40	*p*-Value
CD14+/CD80+/Glut+ cells: %, mean ± SD	56.7 ± 12	28.6 ± 10.8	23.7 ± 7.9	0.038 ^a,b^
CD14+/CD163+: %, mean ± SD	34 ± 11	52.7 ± 15	49.9 ± 11.8	0.047 ^a,b^
CD14+/CD80+/HLADR+: %, mean ± SD	51.9 ± 11	21.9 ± 12	31.5 ± 18.6	<0.001 ^a,b^
CD14+/CD163+/CD206+: %, mean ± SD	21.9 ± 11.6	40.3 ± 15.4	39.7 ± 15.2	0.003 ^a,b^
CD14+/CD80+/Arg1−: %, mean ± SD	58.7 ± 4.3	29.6 ± 10.3	30.7 ± 9.8	0.0413 ^a,b^
CD14+/CD163+/Arg1+: %, mean ± SD	22 ± 6.2	37.3 ± 11.5	36.4 ± 12.9	0.001 ^a,b^
CD80+/CD163+ ratio: %, median ± SD	2.5 ± 0.7	0.8 ± 0.3	0.9 ± 0.4	0.019 ^a,b^
**Lymphocyte subpopulations**				
CD3+: %, mean ± SD	39.8 ± 17.1	35.8 ± 13.5	31.5 ± 11.6	0.678
CD3+/CD4+: %, mean ± SD	22.5 ± 11.8	25.1 ± 3.3	35 ± 9.9	0.447
CD3+/CD8+: %, mean ± SD	33.3 ± 19	20.6 ± 8.5	22.9 ± 7.9	0.444
CD4/CD8 ratio: mean ± SD	0.8 ± 0.5	1.4 ± 0.6	1.4 ± 0.4	0.016 ^a,b^
**mTOR/AKT intracellular expression**				
CD14+/mTOR+: %, mean ± SD	22.3 ± 12.1	48.2 ± 12.7	41.6 ± 13.6	0.001 ^a,b^
CD14+/AKT+: %, mean ± SD	21 ± 4.2	51 ± 16	49.6 ± 4.5	0.002 ^a,b^
EPCAM+/mTOR+: %, mean ± SD	20.1 ± 7.2	52 ± 12.6	47.5 ± 13.4	<0.001 ^a,b^
EPCAM+/AKT+: %, mean ± SD	23.6 ± 6.2	51.4 ± 10.1	51.5 ± 6	<0.001 ^a,b^

Abbreviations: TAM, tumor-associated macrophage; HGS-OC, high-grade serous ovarian cancer; SD, standard deviation. ^a^ significant difference (*p* < 0.001) between patients with papillary HGS-OC and patients with endometriosis; ^b^ significant difference (*p* < 0.001) between patients with papillary HGS-OC and patients with other histotypes.

**Table 3:** Circulating and ascitic fluid inflammation levels, oxidative stress, and iron metabolism parameters.

Circulating Parameters: Mean ± SD (Range)	HGS-OC No. 178	Other Histotypes No. 20	Endometriosis No. 40	Controls No. 150	*p*-Value
CRP, mg/L	85 ± 56 (7–200)	17 ± 5 (2–23)	2.1 ± 0.9 (0–4.5)	1.5 ± 0.2 (0–5)	<0.0001 ^a,b,c^
Fibrinogen, mg/dL	484 ± 197 (340–758)	350 ± 68 (243–480)	280 ± 120 (140–340)	250 ± 90 (180–300)	<0.0001 ^a,b,c^
IL-6, pg/mL	80 ± 53 (10–299)	31 ± 13 (13–89)	2.4 ± 1.3 (0–4)	0.5 ± 0.1 (0–1)	<0.0001 ^a,b,c^
ROS, FORT Units	457 ± 69 (369–600)	387 ± 65 (236–430)	291 ± 185 (150–350)	200 ± 80 (160–250)	<0.0001 ^a,b,c^
Hepcidin, ng/mL	77 ± 25 (32–125)	33 ± 13 (18–70)	19.13 ± 14 (0–24)	17 ± 11 (3–20)	<0.001 ^a,b,c^
Serum iron, g/dL	31.6 ± 27 (5–172)	84.3 ± 55 (11–171)	65 ± 29.7 (40–171	54.5 ± 25 (40–180)	<0.0001 ^a,b,c^
Ferritin, ng/mL	505 ± 277 (22–1034)	113 ± 91 (35–288)	203 ± 89 (40–250)	80 ± 48 (5–148)	<0.0001 ^a,b,c^
Transferrin, ng/mL	173 ± 35 (115–269)	183 ± 35 (109–298)	171 ± 27.6 (100–250)	160 ± 35 (100–360)	N.S.
**Ascitic Fluid Parameters:** **Mean ± SD (Range)**	**HGS-OC** **No. 178**	**Other Histotypes** **No. 20**	**Endometriosis** **No. 40**	**Controls** **No. 150**	***p*** **-Value**
CRP, mg/L	19.1 ± 7.2 (2.5–38)	14.1 ± 5.8 (4.1–30.2)	0.9 ± 0.4 (0.03–1.8)	0.5 ± 0.1 (0–1.5)	0.045 ^a,b,c^
IL-6, pg/mL	1081 ± 495 (100–1900)	69 ± 37 (6–169)	0.7 ± 0.3 (0.1–3.4)	1 ± 0.2 (0–3)	<0.0001 ^a,b,c^
ROS, FORT Units	469 ± 114 (302–600)	350 ± 190 (202–464)	540 ± 147 (160–800)	80 ± 40 (10–160)	0.047 ^a,b,c^
Hepcidin, ng/mL	94 ± 34 (38–210)	37 ± 24 (12–80)	8.1 ± 2.1 (1.9–21)	1.4 ± 0.5 (0–2)	<0.0001 ^a,b,c^
Free iron, g/dL	25 ± 15 (4–57)	45 ± 21 (13–99)	73.6 ± 45 (21–168)	48.3 ± 30 (20–60)	<0.0001 ^a,b,c^
Ferritin, ng/mL	1005 ± 359 (71–1500)	395 ± 153 (115–469)	590 ± 184 (10–750)	94 ± 57 (10–150)	<0.0001 ^a,b,c^
Transferrin, ng/mL	141 ± 45 (35–192)	151 ± 29 (129–193)	155 ± 65 (89–263)	200 ± 40 (150–300)	N.S.

Abbreviations: HGS-OC, high-grade serous ovarian cancer; CRP, C-reactive protein; IL, interleukin; ROS, reactive oxygen species; FORT, Free Oxygen Radical Test; SD, standard deviation. ^a^ significant difference (*p* < 0.001) between patients with HGS-OC and patients with endometriosis; ^b^ significant difference (*p* < 0.001) between patients with HGS-OC and patients with other histotypes; ^c^ significant difference (*p* < 0.001) between patients with HGS-OC and healthy patients; N.S., not significant.

**Table 4:** Correlation analysis between macrophage polarization (M1/M2 ratio) and ascitic levels of parameters of inflammation, oxidative stress, and iron metabolism in patients with endometriosis and ovarian carcinoma.

Macrophage Polarization and Ascitic Inflammation Levels, Oxidative Stress, and Iron Metabolism in Patients with Endometriosis and Ovarian Carcinoma						
	Patients with Endometriosis		Patients with HGS-OC		Patients with Other OC Histotypes	
**Parameters**	Coefficient (CI 95%)	*p*-Value	Coefficient (CI 95%)	*p*-Value	Coefficient (CI 95%)	*p*-Value
IL-6	0.8096 (−0.6827–0.9958)	0.1904	0.8096 (0.2827–0.9958)	0.015	−0.2154 (−0.9224–0.8233)	0.7279
CRP	0.4274 (−0.0571–0.0945)	0.1657	0.4274 (−0.1941–0.8041)	0.1657	0.3220 (−0.7826–0.9378)	0.5972
Hepcidin	0.8542 (−0.5971–0.9969)	0.1458	0.8542 (0.5971–0.9969)	0.0380	−0.3598 (−0.9428–0.7655)	0.5520
Ferritin	0.02186 (−0.7434–0.7624)	0.9629	0.6164 (0.0263–0.888)	0.0434	0.8062 (−0.6878–0.9958)	0.1938
Free iron	−0.5033 (−0.7304–0.7741)	0.0415	0.462 (0.2757–0.6522)	0.0630	−0.8977 (−0.4826–0.5378)	0.2905
ROS	0.5762 (−0.4421–0.9456)	0.5762	0.6726 (0.1220–0.9067)	0.0233	0.6869 (−0.4960–0.9770)	0.2002
**ROS Levels and Ascitic Levels of Inflammation and Serum Metabolism Parameters in Patients with Endometriosis**						
	Patients with Endometriosis		Patients with HGS-OC		Patients with Other OC Histotypes	
Parameters	Coefficient (CI 95%)	*p*-Value	Coefficient (CI 95%)	*p*-Value	Coefficient (CI 95%)	*p*-Value
IL-6	0.3446 (−0.8715–0.5516)	0.4490	0.3554 (−0.1243–0.4323)	0.0279	0.01634 (−0.6732–0.6549)	0.9667
CRP	0.3920 (−0.2347–0.7885)	0.2076	0.3274 (−0.0941–0.6041)	0.3567	0.2169 (−0.8228–0.9226)	0.7261
Hepcidin	−0.0814 (−0.7073–0.6161)	0.8352	−0.08895 (−0.6539–0.5397)	0.7948	−0.3598 (−0.9428–0.7655)	0.5520
Ferritin	0.4913 (−0.2003–0.8561)	0.1493	0.5574 (0.03803–0.8396)	0.0584	0.5395 (−0.8756–0.9882)	0.4605
Free iron	0.8232 (0.4018–0.9569)	0.0034	0.4491 (0.3074–0.9253)	0.0510	0.4977 (0.1826–0.5378)	0.0591

Abbreviations: HGS-OC, high-grade serous ovarian carcinoma; OC, ovarian carcinoma; IL, interleukin; CRP, C-reactive protein; ROS, reactive oxygen species. Results are considered significant for *p* < 0.05.

**Table 5:** Regression analysis between levels of ROS and parameters of inflammation and iron metabolism in the peritoneal fluid of patients with endometriosis.

Parameters	Coefficient	Confidence Interval CI 95%	*p*-Value
IL-6	−15.4377	−63.7764–32.9009	0.4490
CRP	4.8884	−3.1948–12.9715	0.2076
Hepcidin	3.5361	−2.60185–8.70908	0.1458
Ferritin	0.1245	−0.05545–0.3045	0.1493
Free iron	2.7808	1.2170–4.3446	0.0034

Abbreviations: CRP, C-reactive protein; IL, interleukin; results are significant for *p* < 0.05.

## Data Availability

The raw data supporting the conclusions of this article will be made available by the authors on request.

## Abbreviations

The following abbreviations are used in this manuscript:

HGS-OC	High-Grade Serous Ovarian Cancer
mTOR	Mammalian Target of Rapamycin
AKT	Protein Kinase B
CRP	C-Reactive Protein
ROS	Reactive Oxygen Species
TAMs	Tumor-Associated Macrophages
OC	Ovarian Cancer
